# Fifth Annual Meeting of the Ohio Dental Society

**Published:** 1871-02

**Authors:** 


### Proceedings of Societies.

### FIFTH ANNUAL MEETING OF THE OHIO DENTAL SOCIETY.

### DISCUSSIONS.

[Continued from January number, page 33.]

Discussion on Dr. Jenning's paper on "Dental Fees." (See December number.)

Dr. J. C. Whinnery heartily endorsed the views set forth in the essay. He denounced the practice of dentists lowering their prices to get business from other dentists. There are dentists, he said, whose mode of getting business seems to be that of underbidding somebody else. In his opinion it is extremely dishonorable for any member of the profession to practice this thing.

Dr. I. Williams is exceedingly pleased with the essay. One point struck him quite forcibly; that is with regard to underrating the work of other members of the profession. It seemed to him to be exceedingly unkind, in examining a filling that has probably been in the mouth for several years, to say that it is a very inferior operation. When we take into consideration the fact that our work was subject to the action of two of the most tremendous forces of nature—mechanical and chemical—it is not right to criticise the work of other dentists, as is sometimes done. He was always glad, when examining the mouth of a patient where operations had been performed by other dentists, to say to them that such a filling was very well done. And when the work was poorly done, he felt better to say nothing about it.

Dr. Spellman thought the dentist ought to be honest enough to tell the patient that such and such work had been bunglingly done, if such was the case.

Dr. Williams: "I do sometimes."

Dr. Spellman liked the portions of the paper he had heard after coming in, very well. He did not think it obligatory on us to be blowing the bellows for other men who do good work, neither should they where there was a failure, be telling it all around, nor tell the patients of it in such a way as to make them dissatisfied with the previous operator. They should be careful to protect the honor and dignity of the profession. If a man should come to him with several operations in his mouth that were entirely worthless, he would be deceiving the patient to permit them to remain so to the utter loss of the teeth, and would be guilty almost equally with the man that made the operations.

Dr. W. H. Sedgewick was pleased with the essay. He often felt that many worthy men in the profession were not properly paid for their services. When men spend time to acquire knowledge and skill to do their work well, they should be properly remunerated for it. He believed the meetings of our Society is fast doing away with the jealous feelings and fault-finding that was prevalent in some localities. Said he, "We should, however, be honest to ourselves, honest to our patients, and, while pointing out any defect in the natural organs of our patients, we should be perfectly free to point out any defects in teeth that have been filled. This, however, could be done in such a way as to cast no reflection upon the profession." He wished they could approach uniformity in their list of prices; as the work of some, however, was undoubtedly worth a great deal more than that of others, there could not be entire uniformity. If they expected to raise themselves in the estimation of the public, they must put a value upon their services.

Dr. G. W. Keely thought it was seldom so short a paper contained so many good points. He concurred with Dr. Whinnery that it was unkind and unjust to underrate the work of others. He thought by so doing they lowered themselves in the estimation of their patients. While he believed it their duty to be honest and inform patients of any defective work they might find in their mouths, yet this could be done without the wholesale condemnation sometimes indulged in.

In regard to fees, he had his regular prices. He thought every dentist should have his minimum prices for different kinds of work, and then difficult operations consuming a great deal of time could be charged for accordingly. Every man should be able to appreciate his work, and where the services rendered were more laborious and lengthy and required more care and skill he should have the courage to charge for it.

Dr. A. A. Blount agreed with the essay in every part. He did not see why they should not charge a fair price for their work as well as lawyers. When extra labor is required in making a filling they should be paid for it.

Dr. N. W. Williams liked the paper quite well. With regard to extracting teeth, he thought it one of the relics of a former age. Every dentist should get rid of it. Another relic of the dark ages, is that of making one set of teeth called "temporary" for the purpose of securing a permanent set—that is, making for ten dollars for the purpose of getting to put in a permanent set. He thought they should charge for every operation whatever it was worth.

Dr. J. C. Whinnery said it had become so prevalent in his part of the country to extract teeth for nothing, that a great many people had received the impression that surgical dentistry was not worth paying for.

An Essay was then read by Dr. W. M. Herriott on "Artificial Dentures." See December number, Vol. 24.

· Dr. H. A. Smith asked if he would recommend any material for a base, the composition of which he did not know?

Dr. Herriott replied that the material he had recommended was offered to them by honorable and honest men and good dentists, and that was enough to convince him that it was worthy of trial by them.

Dr. A. A. Blount desired to add his testimony to that of Dr. Herriott's, in regard to the value of Watt & Williams' base. It could be made almost as light and thin as gold plate. It was not so heavy as continuous gum-work. He had been experimenting with it some, and was satisfied that there was no cheap base that was equal to it.

Dr. H. A. Smith inquired of Dr. Blount if he knew what it was made of.

Dr. Watt remarked that there were but three persons in the world that know what it is composed of, and *for the present*, at least, none others would know. There was not a man present, unless one in the adjoining room, that knew what the artificial teeth they used were made of. The objection, therefore, that they did not know what this base was composed of, was without weight.

Dr. W. M. Herriott again spoke in commendation of the new base. He had some experience with it. His patients who had worn it spoke of it as being comfortable. It could be made but little heavier than gold plate, and he could make a more perfect fit with it than he could with gold plate. As a cheap base he had never found anything its superior.

Dr. B. T. Spellman said such were the prejudices of many persons that it was difficult to introduce anything of this kind. It would be remembered that he had been no friend to rubber for many years; he had written the first paper condemning rubber which was read before the American Dental Association. He had been one of the first in Ohio to go into rubber work, but became convinced that it was not a good base. He was using the base here referred to, but not very heavily as he was not doing much artificial work: but he was experimenting with it, and would continue to do so. The trouble was that the public wanted a cheap denture to fill all the requirements of the best work.

This was entirely wrong. This base was presented to the profession as a cheap material, and the important question is, is it destructible or not? There is no advantage in a cheap base as destructible and injurious as rubber—perhaps nothing had been used for this purpose so worthless as rubber. It works destruction to the mouth, and is not lasting itself. This material he knew was destructible to some extent; a slow oxidation goes on in all these metals. The gentlemen that are advocates of it ought not to deny that fact. There is a slow oxidation continuously going on, but that oxidation, so far as he could discover, worked but little injury, and he preferred it to any cheap material that has been offered to the public; he was using it and expected to continue to use it, and rose to give his influence in its favor. He thought it infinitely better than rubber, and the plates much more durable; some might suppose he did not use the rubber because he had no license to do so, but every one present knew he could get a license any time he chose, but he would not take one as a gracious gift. [Applause.] No piece of rubber work should ever go up in his office again with his knowledge. [Renewed applause and cries of "good."]

Dr. A. A. Blount thought the weight of this material was no objection; a denture of sufficient weight is much firmer and enables the patient to masticate much better than with a light one.

Dr. H. A. Smith had every confidence in Dr. Watt's material; the question with him was, whether under the code, they had not a right to know what it is. He was in favor of patents himself, and in this particular differed from the code.

Dr. J. H. Warner, of Michigan, on invitation, addressed the

Convention. He returned his thanks to the Society for the kindness extended to him by them. The remarks of the gentlemen on this subject had pleased him very much. He concurred in the opinion that these cheap materials for a base should only be used as a last resort, but he thought that last resort had probably come. Many patients were not able to pay for the more expensive dentures of gold or continuous gum work, so that a cheaper one is demanded. He had discarded rubber, and would go to mending shoes, or do anything else for an honest living, before he resort to this useless rubber again. [Applause.]

He had been using the Weston metal, and preferred it to rubber, and would go before his patients to-day and attempt to build up a reputation as a skillful dentist upon that, a good deal sooner than with rubber. He believed Drs. Watt and Williams had invented something far superior to Weston's metal, and would investigate the matter. He thought probably some member of this Society would yet invent something superior to Watt & Williams' material. He hoped before the next annual meeting of this Society there would not be a man in the United States that would use rubber plates. He would that the last rubber plate had been made, and the practice of making them were numbered among the dark ages of the profession.

Dr. Watt said, as remarked by Dr. Smith, there is a radical difference between an alloy and a mechanical mixture of metals. An alloy, as he understood it, was a compound of two or more metals chemically combined with each other, while a mechanical mixture is something vastly different. In his younger days he had fallen into the mistaken notion given by many authors, that alloys would not resist chemical action as well as the metals of which they were composed. His observation, at an early day, led him to see that this was an error. Every one there knew well, that brass resists chemical action better than either of the metals of which it is composed.

Dr. W. P. Horton thought it well to have some good cheap material for a base, for though gold answers the purpose quite well, not a tithe of the people are able to pay for it. He had a great deal of sympathy for the poor who are not able to pay the higher prices demanded for the best material.

### EVENING SESSION.

Discussion on a paper read by Dr. C. R. Butler, on "The Discipline of the Head, Heart, and Hand." (See December number.)

Dr. Sedgewick heartily endorsed the sentiments of the paper. He had sometimes felt like expressing himself as Dr. Berry, of Cincinnati, had; that he would never take another student, but he would like to see men like Dr. Butler, and others he might name, take young men and educate them for dentists. It was their duty as dentists, to point out to young men who contemplated entering the profession, the great responsibility of the profession, the difficulties to be surmounted, and the education necessary to make a successful operator; and that he should not only possess a fine education and have a studious mind, but should also in a great degree, possess the qualities of the artist, and if he has not these they should discourage him.

Dr. W. M. Herriott was pleased with the idea advanced in the essay, that there was something due to the operator as well as to those operated on. Many in the profession he thought, did not rightly appreciate this. They went about seeking business in a way that showed this, peddling themselves as it were—going before a community and begging people to come to them for operations. He believed each operator should feel that it was as much a privilege to the people among whom he was living and for whom he was operating, to have the privilege and benefit of his operations, as it was to him to receive the amount of money they paid him for his services. Just as soon as the profession comes up to this point, and cease offering to do the *very best* work at the *very lowest prices*, or agree to warrant their work, or do this, that, or the other—and then come up and thank the people for their appreciation of their work—as soon as they reach this independent standpoint, the people will appreciate them as they desire to be appreciated.

In reference to educating students for the profession, he thought there had been too much laxity. In talking to young men who thought of entering the profession, he had advised them to give at least three year's study to the profession, before they should consider themselves able to operate for themselves. If all would do this, it would be better for the profession and infinitely better for the people.

Dr. J. Taft said the paper embraced a subject that was very interesting and important to every one of them. Under the views presented they might discuss almost any of the duties devolving upon them as professional men; their duties were exceedingly diverse. First there was the preparation and attainments necessary on the part of the practitioner. He should place himself in a proper position on this point. All of them should give great attention to this, that they may exert a proper influence upon those who propose to enter the profession. No honest, upright young man will seek to enter the profession in any other than a proper way. No one when he starts out, wishes to become a second-rate, or third-rate dentist. In some cases young men will apply to one and another to be received as a student, but will not be received, and finally will be found under the instruction of some one wholly incompetent to advise them, to say nothing about teaching them; men whose influence would divert them from a right course. If the members of this Society all did their duty as they ought, this would be otherwise with those who come into the profession. If they could not receive students themselves, they should advise them as to what they ought to do. If they could not receive them, they should send them where they will be in charge of good men, surrounded by proper influences. Young men had frequently come to him, who had been in the office of some dentist for three or four years, and yet they did not know the first letter of the elementary principles of a professional education; had never perhaps, opened a book on physiology or pathology, and had scarcely any idea of the definition of these words. Some who are so poorly qualified, knowing nothing of the fundamental principles of dentistry, think themselves well prepared to go out and engage in its practice. Of course these persons had fallen into wrong hands. It was the duty of members of the profession to see that students accomplished all that the profession demands of them. If every man among them who is a good practitioner and possesses a good degree of attainments in the profession, would take the right stand and act up to his conscience and knowledge in this matter, a wonderful revolution in this respect would be accomplished in a short time. It is competent, said he, for our Society to make its influence felt in this matter. Where is the society that has given its influences to our colleges and done what might have been done in this matter? Our colleges are very far below the demands of the times in regard to the mode of their teaching and their efficiency, and why? Because of the facts mentioned. The standard in the colleges is too low everywhere. Why is it that men who can not write a sentence in the English language correctly, can enter and graduate in one session? If we would prevent men thus being sent out half-fledged, we must come out and take a higher standard. We are all at fault in this matter, somewhat necessarily so because the profession has been lying asleep in the matter.

He thought they should be careful in the selection of men for the profession, taking only those who possess natural ability and the proper acquired ability, and then have them put into their schools for two or three years. If this was done a great change in this respect would soon be wrought. Their own State Society, he was proud to say, has made a move far beyond that of any society in the country; that was with regard to the procurement and carrying out of the provision of the law they now have. They were now standing head and shoulders above other societies all over this country, and the profession in Ohio has a reputation it nowhere else possesses, because of this. If this Society would take a still higher stand with regard to education, it would have a greater influence.

Dr. C. R. Butler said the Society did not stand here as a school to teach all creation. They stood here as a legislative body of the profession in the State of Ohio. The principles that underlie the practice, and that pertain to the profession, should be taken up and discussed before the Society. There was much grumbling all over the country that they were not recognized as professional men. Just as soon as they deserve it they will be properly recognized, and until they are worthy they need not expect the people to accord to them the respect and honor that should be accorded to such a profession.

The work of citing cases and discussing the simple every day practice, he thought belonged more properly to the local societies. This State Society possesses a power that no other society under the light of the sun possesses; even the National Association has no more power than this. It had not done so much for the elevation of the profession as the Ohio State Dental Society has done.

In regard to their schools, if they do not amount to much it was the fault of members of the profession. They will amount to all we demand of them. The speaker closed with a reference to the Ohio Dental College, and the need of more suitable buildings for the College. He remarked if each of the members of the profession in Ohio would give but five dollars, for three years, they could put up a building in Cincinnati, or Columbus, or any other desirable point, that would be an honor to the profession all over the land, and more especially to them. [Applause.]

Dr. H. A. Smith thought, at the present time it was more desirable to raise the means to pay off the indebtedness of the College. Though the amount was not large, still the debt should be canceled, and this should be done before they talked of a new building.

Further remarks were made in regard to the Ohio Dental College, by Drs. Keely, Taft, Watt, Horton, and others.

Dr. Decamp, of Mansfield, said one remark in the essay struck him rather forcibly; that was to keep your sign in your office. Heretofore many had been in the habit of placing their signs prominently out and warranting to give perfect satisfaction or no pay, &c. This we see in newspapers, on handbills, everywhere. But there is progress; and evidences of it were seen in their Society. In reviewing the work of the profession for the last twenty years, he could see unmistakable signs of progress. Twenty years ago very few were making any active efforts to educate the profession. He would like to be a young man again, to enjoy the advantages the young men now enjoy.

After a few further remarks, the paper was, on motion, referred to the Committee on Publication.

The discussion of the fourth subject was next in order, viz.:

### ANÆSTHETICS IN DENTISTRY, AND MODERN SURGERY.

Dr. Watt by request, after a few prefatory remarks, said a mistaken notion prevailed pretty generally in regard to anæsthetics; most persons supposed anæsthesia meant about the same thing as unconsciousness, or about the same thing as being deranged. During the last two or three years he had had opportunity to talk with many members of the dental profession, and they nearly all labored under this mistake; that the terms unconsciousness and anæsthesia were synonymous. And if they wish to express how thoroughly successful they have been in the use of anæsthetics, they will tell you how utterly insensible, or crazy, the patient has become when they administered them. They deem it a great success if they can make the patient so crazy that, in his sane moments, he remembers nothing about the operation, the cutting, crashing, &c. Said he, if you have complete success in using an anæsthetic, you have the patient as conscious as we each one are conscious now. And the sense of touch is as distinct and the patient as well aware of what is done as you would know it now—knows just where the knife is going, how deep it goes, and all about it. The patient, in such a condition, will not be afraid; not only is he conscious, but conscious that he will not be hurt.

This was something that was not generally believed. He did not say that an operator could not have perfect anæsthesia with some other physiological, or pathological, or artificial conditions at the same time. If there is unconsciousness with the anæsthesia, that ought to be regarded as a failure, but not a serious failure if that is all we have. But when you are undergoing a terrible operation, the satisfaction of knowing that you are alive, and what is being done to you, and supervising the operation yourself, according to your own judgment, is a great luxury.

The pleasure of this consciousness is something that never ought to be taken away when it can be avoided.

If, in addition to unconsciousness, delirium is induced, the evil effects are still greater. He would not claim that it is possible to have absolute success in the present condition of things, but it is possible in a very large proportion of instances; and if the public mind were educated, and the profession educated up to the belief that it is not necessary in operations to produce unconsciousness, then it would be more easy to have success. Patients have been taught that they must be rendered unconscious, that if they are not, they will suffer pain. Because of this, it may be difficult in some cases, to have perfect anæsthesia with consciousness.

He had used chloroform something over thirteen hundred times. In almost all cases where the patients had confidence in him, and were let alone by others, he had no difficulty in having perfect success. The patients were able in such cases to describe the operations as perfectly as he could.

He thought chloroform had more tendency to produce delirium than nitrous oxyd, but carbonic acid, and especially carbonic oxyd, has a great tendency to produce delirium, and if, from any cause you retain the carbonic acid and throw it back into the system, delirium will ensue.

The doctor then related in detail, two or three cases in which he had administered chloroform. In one case he mentioned that he administered it according to the favorite plan of a surgeon; that was to take a very thin crash towel, that was easily breathed through, and spread it over the face, upon which a drop was let fall at a time. That is a very good way to ensure getting atmospheric air with the chloroform, but the expiration is not made with sufficient force to carry off the carbonic acid from the air-cells.

He had probably seen one hundred and fifty cases in which it was used in that way, but he had never seen one case in which there was not some signs of delirium. He doubted if ever any of them saw a case treated in that way where any considerable amount of chloroform was given, that the patient was not to some extent rendered delirious. In a large majority of the cases where chloroform proves fatal, the patients are killed by carbonic acid. Another very considerable portion are probably killed by strangulation.

One reason why there seems to be a large proportion of fatal cases of this kind among dentists is, because they administer chloroform in so many more cases than other surgeons do. The percentage of deaths is not near so large as is sometimes supposed to be the case.

Another point to which he would call attention is this: whenever there is a darkening of the complexion in administering either chloroform or nitrous oxyd, there is something wrong. Neither one has a tendency to darken the blood corpuscles at all. Both have a tendency to render them a little more florid, but if in the administration of either one there is the least sign of darkening the complexion, you are smothering your patient. Many a one has thus been smothered, that has not been anæsthetized at all.

If there is any sign of suffocation, heavy breathing, or anything that indicates that the patient is not breathing perfectly free and easy, throw the exit valve open, and let the patient breathe the atmospheric air rapidly, until the excess of carbonic acid is pumped out. Let in gradually the air until the patient breathes the nitrous oxyd with straight-forward, deliberate breathing, by that time the excess of carbonic acid has been forced out of the blood, and is no longer annoying; further on in the process there is scarcely any carbonic acid given off.

Dr. Keely inquired of Dr. Watt if in the cases he had detailed, he did not think he used some other agent?

Dr. Watt replied that the probability why he succeeded, was because he *did not* use any other agent than the one he had mentioned.

Dr. Keely said it was his opinion that Dr. Watt had used some other agent, one that had been known a good many years—mesmerism. He believed if it had not been for that power exerted by the doctor over the patients in the cases related, he would not have been so successful. When that influence is brought upon the patients and they are thoroughly under its influence, an arm could be cut off without pain, not only when they are in deep sleep, but when as wide awake as any person present. He related a case in point, that occurred in Frankfort, Kentucky, in 1847. A lady who was to have some teeth extracted was thrown into a magnetic state. An old-fashioned congress knife ground down to a point, was used to cut around a bicuspid root that was difficult to get at, and though three-quarters of an hour was spent in the operation, when asked if it hurt her, she said no, that she felt more like going to sleep than anything else.

Another case was given where the patient was a girl, and the operation performed before a large audience in St. Louis, with like satisfactory results.

After a few remarks by one or two other speakers, the meeting adjourned until 9 A. M. next day.

Discussion on "The Use of Mallets in Excavating and Filling Teeth."

Dr. Harroun said he could not get along without the use of the mallet, in some form in filling. He was now using a bell-shaped mallet, with two faces. He had dispensed with all lighter mallets, using now a five-ounce mallet, and knew he could control it better than a light one. With it he could get just as light or as heavy a blow as he wanted.

Dr. Spellman was, as he expressed himself last year, in favor of the mallet. He could not think of filling teeth without it. He was fully convinced that our best operations are with the mallet. It did not seem to him that he could return to the old practice. A friend of his had said to him that he believed he would leave the profession if he had to dispense with the use of the mallet. He did not know that he was so enthusiastic in its favor as that, but he could not do without it. He used a six-ounce mallet now; had used an eight-ounce mallet, but found it too heavy; had also used one weighing four ounces, which was not quite heavy enough, and he had now settled on a six-ounce one. It probably took longer to make a filling with a mallet, but the work was better done and gave better satisfaction. He thought that that mode of manipulation that required the most time, as a general rule, will produce the best result.

Dr. Rosson was in favor of the mallet; had experimented with different mallets and now settled down on a four-ounce mallet. He used an assistant in all his operations in filling teeth. He did not use the mallet entirely; used some hand pressure in introducing the gold, and the mallet in filling and condensing.

Dr. Whinnery could not think of giving up the use of the mallet. He could make a better filling with it than he ever could before without it.

Drs. Lilly and Moffatt both expressed themselves in favor of the mallet.

Dr. I. Williams thought the use of the mallet had become so well established that arguing in its favor, seemed to him very much like a religious society he heard of some time ago offering a resolution in favor of Sabbath Schools, and urging the propriety of establishing them.

He had used a lead mallet, but did not like it very well. Was now using a wooden mallet and thought it better in every respect.

Dr. Kelsey favored the use of the mallet, but thought there was, perhaps, such a thing as an abuse of the mallet. Those not accustomed to using it might do injury with it. He had seen a case of this kind, and had in his possession a couple of molar teeth that had been absolutely split open by a mallet. A Methodist minister had come to him who had the operation performed in an adjoining county. He found the teeth split apart nearly a sixteenth of an inch. He would say nothing disparagingly of the proper use of the mallet, but simply expressed the opinion from what he had seen, that there might be an abuse of it. [The teeth mentioned by the doctor were then given by him to the members of the Society for examination ]

Dr. Herriott desired to hear from those who used the automatic plugger, with regard to their success or failure. It had been said by some that they thought it impossible to fill teeth as well by hand pressure as with a mallet. He had seen teeth thus filled that had remained in an excellent state of preservation for many years. He had a tooth in his own mouth, filled by Dr. Morehead nearly twenty years since, which was now as perfectly solid as when he filled it. He stated these facts, not that he did not consider a mallet a good thing; he could not make a good crown filling without the mallet.

Dr. J. Taft asked Dr. Kelsey whether he knew positively that the teeth he had exhibited, had been split open with a mallet while in the mouth of the patient.

Dr. Kelsey said he simply had the word of the man himself who had the operation performed. He supposed him to be a man of veracity; as he is a Methodist preacher, he thought he ought to be. When he came to him the fillings had worked out; he said they had been put in with tin foil and that the teeth had absolutely been split open with the mallet. He could not conceive how it was done; but supposed the cavity was too small for the instrument, and that the instrument had been wedge-shaped, and that by striking upon it, the teeth had been split open. The dentine seemed firm and in good condition, and there was no necrosed condition of the teeth. The man came to him quite loth to lose his teeth, but was suffering very severely and there seemed to be no remedy but to extract them.

Dr. A. A. Blount desired to add his testimony in favor of the mallet. It seemed hardly necessary to say anything on the subject. Teeth could be filled so much better with the mallet than by hand pressure, that there was no comparison. He did not think it possible that a tooth such as described by Dr. Kelsey, could be split by using a mallet, unless it had been by a sledge-hammer.

Dr. Harroun said he had used an automatic plugger until he had become familiar with its use. Whenever his assistant was engaged so he could not help him and he could not use the ordinary hand mallet, he used the automatic plugger.

He had become so familiar with it that he could control it perfectly, and could get any pressure he desired; and could save one-third of his time in doing the work, and could put it in just as solid as with a hand mallet.

Dr. Spellman said the tooth he had from Dr. Kelsey was very little decayed, and if such a tooth was split in the mouth with a mallet, the patients in that section must be a different class of people from those found elsewhere, or they could not endure any such thing. The force that would be required to split such a tooth no man or woman would endure.

Dr. C. R. Butler thought it almost useless to talk about the value of the mallet, so well established was that fact. It might probably be abused by improper use, but to speak of splitting teeth like those exhibited by Dr. Kelsey, in filling them seemed almost impossible.

Dr. Spellman inquired if the cracks were in them before they were drawn?

Dr. Kelsey: They were lying open so that I could put the instrument in the fissure.

Dr. Spellman: Did you cut around the tooth to extract it?

Dr. Kelsey: No, sir, not at all, there was no necessity for it.

Dr. Spellman remarked that there was no evidence that ever a drop of blood had been upon it. This was something rather strange.

Dr. Kelsey: I have a plan for extracting teeth so quickly that they don't bleed.

Dr. Rehwinkle thought it possible that, by an unguarded movement, the instrument, either in excavating or filling, may have entered as a wedge, and split the tooth. That only however, shows an abuse of the mallet.

He feared in their discussions they were apt to manifest a want of liberality, and were too apt to become too enthusiastic in favor of their own opinions, and too free to condemn the opinions of others.

Dr. Rehwinkle then gave a brief description of the use of the mallet, dating its first use back to 1853. His now deceased predecessor, at that time, used a little steel hammer in filling teeth. Shortly after this, Dr. Atkinson set forth the extraordinary benefit of the mallet. He could not see how dentists could get along without the mallet. They could make good fillings with the mallet where they could scarcely make them at all by hand pressure. With regard to wood, lead, or steel mallets, he thought more depended upon the skill of the hands that use them, than upon the material of which the mallet is made. If the testimony of patients could be obtained, he thought those in favor of any one of these kinds of mallet would about equal those in favor of either of the others.

Dr. Horton said it would be almost impossible for him to perform many operations without the use of it. He had found it particularly useful in performing operations on children whose teeth were very sensitive. The mean motion, he remarked, of a six-ounce mallet was not over one-sixteenth of an inch in cutting down the teeth, in which operation he used a series of chisels; whereas the mean motion of the hand in excavating, is from an inch to an inch and a half. With the mallet, an operator could chisel out in a few minutes what it would take an hour to do with the hand, with an ordinary excavator. Old people he found, objected more to the use of the mallet than children.

